# Nano-Microemulsions of CaCO_3_-Encapsulated Curcumin Ester Derivatives With High Antioxidant and Antimicrobial Activities and pH Sensitivity

**DOI:** 10.3389/fvets.2022.857064

**Published:** 2022-08-11

**Authors:** Lian Wang, Xuefei Wang, Zhiwei Guo, Yajuan Xia, Minjie Geng, Dan Liu, Zhiqiang Zhang, Ying Yang

**Affiliations:** ^1^College of Veterinary Medicine, Inner Mongolia Agricultural University, Hohhot, China; ^2^College of Veterinary Medicine, Henan University of Animal Husbandry and Economy, Zhengzhou, China; ^3^Inner Mongolia Autonomous Region Comprehensive Centre for Disease Control and Prevention, Hohhot, China; ^4^Baotou City Primary Health Service Guidance Centre, Baotou, China; ^5^Bayannaoer City Centre for Disease Control and Prevention, Bayannaoer, China

**Keywords:** antioxidant activity, pH response, antimicrobial property, CaCO_3_@Cur-FA, biological activity

## Abstract

In this study, we synthesized nano-microemulsions of calcium carbonate (CaCO_3_)-encapsulated curcumin (Cur)-Ferulic acid (FA) ester derivatives of diverse mass ratios by using the solution casting approach. The structures, antioxidant and antimicrobial activities, physical properties, and potential of hydrogen (pH) sensitivity of these products were examined. Compared with microparticles of CaCO_3_, those of CaCO_3_@Cur-FA exhibited excellent antimicrobial and antioxidant properties. Response to pH was indicated through the release of Cur-FA from CaCO_3_@Cur-FA in solutions having different pH values. The results demonstrated that Cur-FA was released more quickly from CaCO_3_@Cur-FA at pH 5.5 than at pH 7.4. CaCO_3_@Cur-FA demonstrated good antioxidant capacities through its ability to scavenge 2,2′-amino-di(2-ethyl-benzothiazoline sulphonic acid-6)ammonium salt (ABTS^+^) and 1,1-diphenyl-2-picrylhydrazyl (DPPH). These activities were three-fold more than those observed in CaCO_3_ microparticle control groups; additionally, the antimicrobial activity against *Aspergillus niger* and *Escherichia coli* increased by 40.5 and 54.6%, respectively. Overall, the microparticles of CaCO_3_@Cur-FA outperformed Cur-FA in terms of antimicrobial properties by inhibiting the growth of certain zoonotic pathogens.

## Highlights

- CaCO_3_@Cur-FA microspheres showed spherical morphology with uniform size.
- CaCO_3_@Cur-FA exhibited excellently antioxidant activity.
- CaCO_3_@Cur-FA exhibited excellently antibacterial activity.
- CaCO_3_@Cur-FA exhibited excellently pH responsivity activity.
- CaCO_3_@Cur-FA could as a new food additive, which applicated in the food industry,

## Introduction

Curcumin (diferuloylmethane; Cur) is a leading curcuminoid extracted from the rhizome of *Curcuma longa* plant (1) and has been used as a traditional herbal medicine across Asian countries. This is because it has antioxidant, anticancer (2), antiviral (3), anti-inflammatory, and antimicrobial properties that help in controlling chronic disorders (4). In addition, curcumin, as a common natural pigment, is extensively used in various food industries such as those of canned food, sauces, and brine products. Cur has high solubility in solvents with lower polarity (for example, ethanol and propylene glycol), and it is deemed to be a lipophilic substance (4). Ferulic acid (FA) is suggested to markedly modulate the incidences of inflammation, oxidation, and metabolic syndrome (5). FA is easily soluble in solvents with high polarity (such as ethanol, water, and methanol) and is deemed to be a hydrophilic substance (6). Lipophilic and hydrophilic phytochemicals are extensively distributed in daily foods (such as plants, fruits, herbs, grains, and vegetables) and exhibit antiviral, antimicrobial, and antioxidant activities when used in combination (7). Based on this interesting finding, we designed a molecule in which Cur and FA were connected via an esterification reaction; this molecule was designated as Cur-FA and could be used as a novel food additive.

At present, nanotechnology has offered measures to cope with diverse technical challenges in various fields such as the food industry (8, 9). Calcium carbonate (CaCO_3_) delivery systems (10) play an important role in various fields including biomedicine, healthcare, and industrial manufacturing (11). They can be used easily and extensively because of their high yield, cost-effectiveness, non-toxicity, biodegradability, biocompatibility, and appropriate decomposition efficiency, apart from their stability even in a basic environment (12). Therefore, CaCO_3_ together with its modified substances could be used as a delivery vehicle and immobilizing carrier for food research, thus reducing the effective dose required (13). Besides, these materials have small droplet sizes, which enhances their antimicrobial bioactivity by allowing their penetration into the cell membrane, resulting in lipid bilayer destabilization.

In the present study, we hypothesized that the nano-microemulsion of CaCO_3_-encapsulated curcumin ester derivatives would have a higher antimicrobial activity against pathogens. This may be because the nano-microemulsion has a small droplet size, which can facilitate its penetration into the microbial cell membrane for destroying its activity and inducing death. This study characterized the nano-microemulsion by using various approaches. Additionally, the nano-microemulsion was examined for its antimicrobial ability against seven zoonotic pathogens (*Escherichia coli, Staphylococcus aureus, Rhizopus*, and *Aspergillus niger*).

## Materials and Methods

Sodium carbonate (Na_2_CO_3_) and calcium chloride (CaCl_2_) were provided by Xiqiao Science Co., Ltd. (Shantou, China). Cur-FA: ^1^HNMR (CDCl_3_, 400 MHz) δ:3.83 (s, 6H), 3.85 (s, 3H), 3.87 (s, 3H), 4.59 (s, 2H), 5.59(s, 2H), 6.31 (d, 1H), 6.79 (d, 2H), 6.91 (d, 3H), 6.93 (d, 1H), 6.99 (d, 2H), 7.06 (d, 1H), 7.10 (d, 1H), 7.11 (d, 2H), 7.20 (s, 1H), 7.23 (s, 1H), 7.27 (d, 1H), 7.48 (d, 1H), 7.60 (d, 3H), 9.5 (s, 2H). HRMS (ESI) calculated for [M + H]+C_42_H_38_O_12_: 734.75 found 734.69.

### Microbial Cultures

Four foodborne pathogenic strains were provided by the Microbiology Laboratory of the National Research Centre (Egypt), which included *E. coli, S. aureus, Rhizopus, A. niger*. Microbial cultures were maintained in a suitable agar medium inclined at an angle of at 4°C (slant culture), which served as the stock cultures. The pathogens were grown in Mueller–Hinton agar (MHA) or Mueller–Hinton broth (MHB).

### CaCO_3_ and CaCO_3_@Cur-FA Microparticle Preparation

In this study, CaCO_3_@Cur-FA microparticles were prepared by a method described previously. Briefly, water: acetone (3:1) solution was mixed with Na_2_CO_3_ (0.5 M) and CaCl_2_ (0.5 M) with Cur-FA1 (0.1 M), Cur-FA2 (0.2 M), or Cur-FA3 (0.3 M) to prepare the stock solutions. The CaCl_2_ solution was mixed with Cur-FA (Cur-FA1, Cur-FA2, or Cur-FA3) in a 50-mL beaker, followed by 10 min of stirring with the 85-1 constant temperature magnetic stirrer (Shanghai Zhiwei Electric Appliance Co., Ltd) to prepare CaCO_3_@Cur-FA microparticles. After the rapid addition of Na_2_CO_3_ solution to the aforementioned mixture, the obtained solution was subjected to 5 min of stirring at 500 rpm at 40°C, followed by centrifugation to collect the products. CaCO_3_ microparticles were synthesized using a similar method without adding Cur-FA solution.

### Characterization of CaCO_3_@Cur-FA Microparticles

The Zetasizer Nano-Zeta potentiometer (Nano ZS90, Malvern, UK) was used to measure zeta-potential and particle size of the CaCO_3_@Cur-FA microparticles. The scanning electron microscope (SEM, Model S-4800 II FESEM, Hitachi, High-Technologies Co., Ltd., Japan) was used to observe the particle morphology.

### pH Sensitivity of CaCO_3_@Cur-FA Microparticles

To assess the pH sensitivity of CaCO_3_@Cur-FA microparticles in a food microenvironment, the CaCO_3_@Cur-FA samples (0.2 mg, 2 mL) were dissolved in phosphate buffered saline (PBS) of various pH (5.5, 6.8, and 7.4) and added into dialysis tubes (MWCO 3500). Thereafter, these tubes were soaked in 10 mL of PBS with corresponding pH (within the centrifuge tube) by constant shaking below 37°C. We took out 1 mL dialysate at pre-determined time intervals to measure the absorption, thereby assessing the amount of released Cur-FA. Then, 1 mL fresh PBS was added to the centrifuge tube.

### Antioxidant Activity Assay

The antioxidant activity of CaCO_3_@Cur-FA was assessed by measuring its ability to scavenge 2,2′-azino-bis (3-ethylbenzothiazoline-6 sulfonic acid; ABTS^+^) and 1, 1-diphenyl-2-picrylhydrazyl (DPPH) free radicals. In brief, 0.2 g CaCO_3_@Cur-FA was dissolved in 2 mL distilled water before the test. Further, 2 mL of the CaCO_3_@Cur-FA supernatant was added into ~2 mL of DPPH methanol solution (0.1 mM) to react for 30 min in dark. Subsequently, the ultraviolet spectrophotometer (Shimadzu, UV-2007, Japan) was used to measure the absorbance (optical density; OD) at 517 nm. The test tube was shaken for ensuring solution uniformity prior to measurements. For the composite film, its ability to scavenge ABTS^+^ free radicals were determined using the modified approach.

The ABTS^+^ or DPPH scavenging ability [R (%)] was determined through (Eq. 1)


(1) $$\begin{array}{r} R\left(\%\right)=\left(A2-A1\right)/A0 \end{array}$$

A0 is the OD value of DPPH (ABTS^+^/PBS) in distilled water;

A1 is the OD value of CaCO_3_@Cur-FA solution in distilled water mixed with the DPPH methanol solution (ABTS+/PBS mixed solution), and

A2 indicates the OD value of DPPH methanol solution (ABTS^+^/PBS mixed solution).

### Antimicrobial Assays

In this study, we adopted agar–well diffusion approach to perform antimicrobial studies. In brief, 1 mL of active strain culture (10^5^ cells /mL) was added to 20 mL MHB (Becton Dickinson, USA) and poured into petri dishes containing MHA. When the agar solidified, wells (diameter, 5 mm) were cut using a sterile borer. Further, 50 μL of the nano-microemulsion or bulk extract was added to each well. The plates were incubated for 2 h under ambient temperature, so that the solutions in the wells diffused into the agar. Moreover, the plates were incubated for 24 h at 37°C. Afterwards, we determined the microbial growth inhibition rate and inhibition zone diameters. Every sample was measured thrice (penicillin G, bulk extract, and nano-microemulsion), and the experiment was performed in triplicates.

Discs containing penicillin G (10 U) and ethanol were used as the positive and negative controls, respectively.

### Statistical Analysis

The differences among the samples were analyzed using ANOVA. Statistical analysis was performed using SPSS software. The significance of difference (*P* < 0.05) was compared by Duncan's multiple range tests.

## Results and Discussion

### Characterization of CaCO_3_ and CaCO_3_@Cur-FA Microparticles

**Figure 1:**
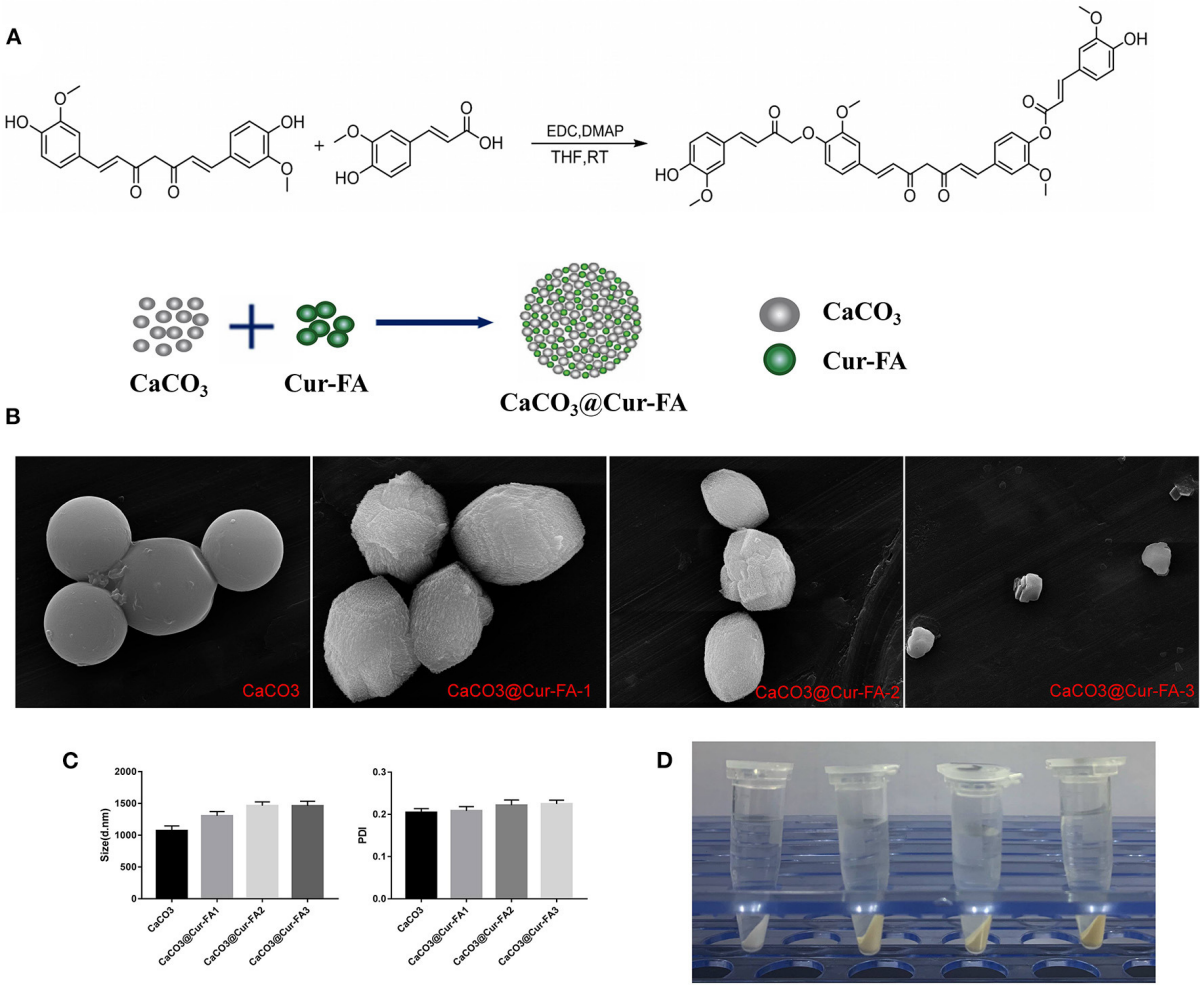
Characterization of CaCO_3_ and CaCO_3_@Cur-FA. **(A)** Synthesis of Cur-FA and CaCO_3_@Cur-FA. **(B)** SEM images of CaCO_3_ and CaCO_3_@Cur-FA. Scale bar = 5 μm. **(C)** Particle size and PDI for CaCO_3_ and CaCO_3_@Cur-FA. **(D)** The images of CaCO_3_ and CaCO_3_@Cur-FA.

This study successfully prepared CaCO_3_ microparticles using the mineralisation approach (Figure 1A). As shown in Figure 1B, the SEM data of CaCO_3_ and CaCO_3_@Cur-FA microparticles clearly revealed the spherical morphology as well as the almost even size distribution. The sample particle size is given in Figure 1C. Table 1 displays the sample zeta potential. The average hydrodynamic sizes of the microparticles of CaCO_3_, CaCO_3_@Cur-FA1, CaCO_3_@Cur-FA2, and CaCO_3_@Cur-FA3 were 1070.1 ± 76 nm, 1303.5 ± 66 nm, 1464.8 ± 58 nm, and 1460.7 ±72 nm, respectively. Thus, the optimal concentration of Cur-FA required for preparing uniform-sized CaCO_3_@Cur-FA microparticles was 0.2 M. The mean size of CaCO_3_@Cur-FA mildly increased relative to that of the unmodified CaCO_3_ microparticles, indicating the successful encapsulation of Cur-FA into the microemulsion. In addition, no droplet aggregation was observed, which indicated that CaCO_3_@Cur-FA maintained its identity in the process of drying. The image of CaCO_3_@Cur-FA microparticles is shown in Figure 1D.

**Table 1:** Characterization of CaCO_3_, CaCO_3_@Cur-FA-1, CaCO_3_@Cur-FA-2, CaCO_3_@Cur-FA-3.

**Nanoparticles**	**Size**	**PDI**	**Zeta (mV)**
CaCO_3_	1070.1 ± 76	0.205 ± 0.009	4.75 ± 0.34
CaCO_3_@Cur-FA-1	1303.5 ± 66	0.208 ± 0.010	−12.34 ± 1.23
CaCO_3_@Cur-FA-2	1464.8 ± 58	0.222 ± 0.012	−13.45 ± 3.08
CaCO_3_@Cur-FA-3	1460.7 ± 72	0.225 ± 0.010	−12.14 ± 1.19

### pH Sensitivity of CaCO_3_@Cur-FA2 Microparticles

**Figure 2:**
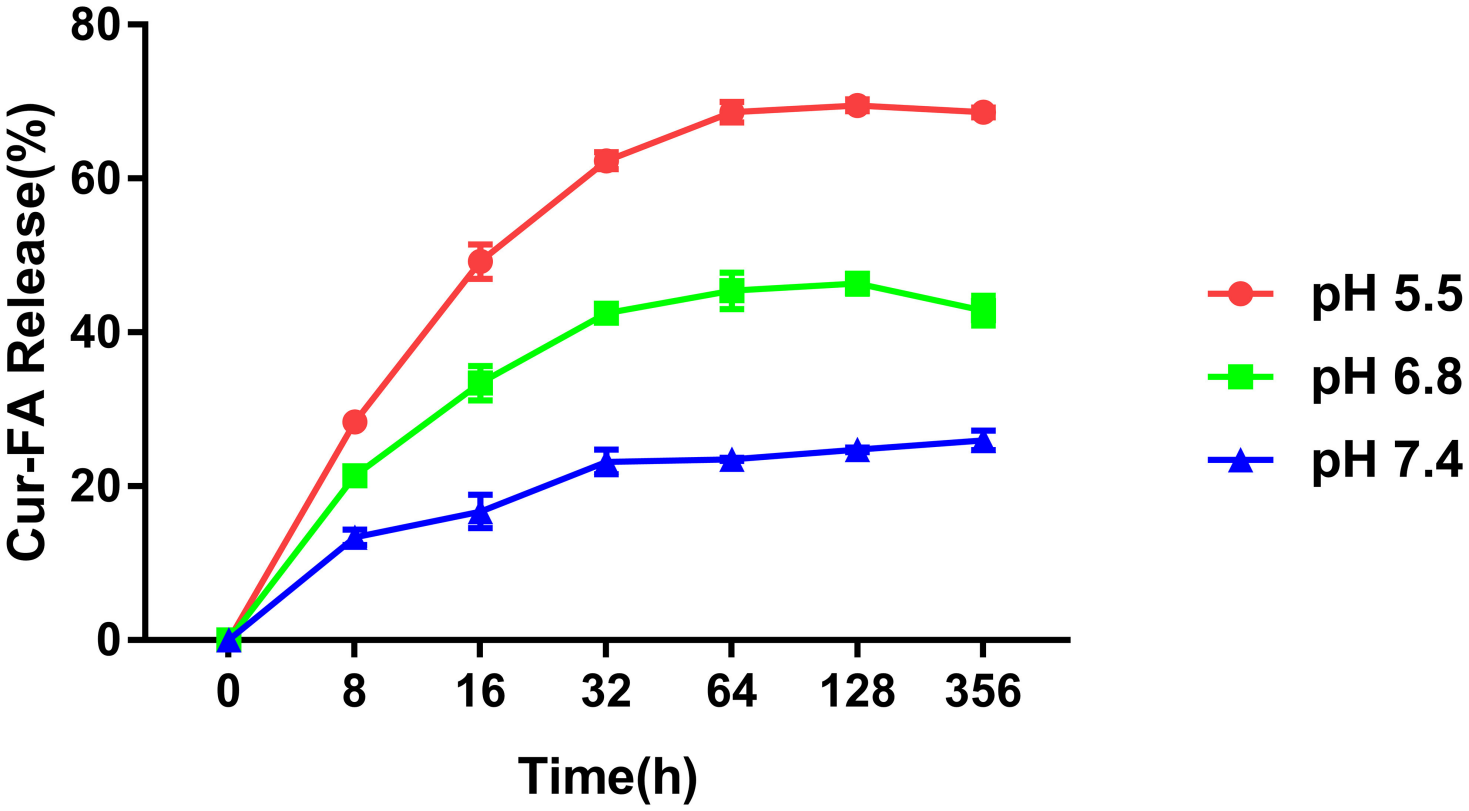
pH sensitivity of CaCO3@Cur-FA. Cur-FA was released from CaCO_3_@Cur-FA at pH 7.4, 6.8, and 5.5.

The pH sensitivity of CaCO_3_@Cur-FA2 microparticles was evaluated in PBS of pH 7.4, 6.8, and 5.5. As shown in Figure 2, CaCO_3_@Cur-FA2 released only 24.5% of loaded Cur-FA2 at pH 7.4 after 128 h. On the contrary, 70.1 and 45.6% of Cur-FA2 was released from CaCO_3_@Cur-FA2 at pH 5.5 and 6.8, respectively, which was associated with CaCO_3_ decomposition under the acidic condition. The results demonstrated that CaCO_3_@Cur-FA could be used as a food additive with a visible pH sensitivity.

### Antioxidant Activity of CaCO_3_@Cur-FA2 Microparticles

**Figure 3:**
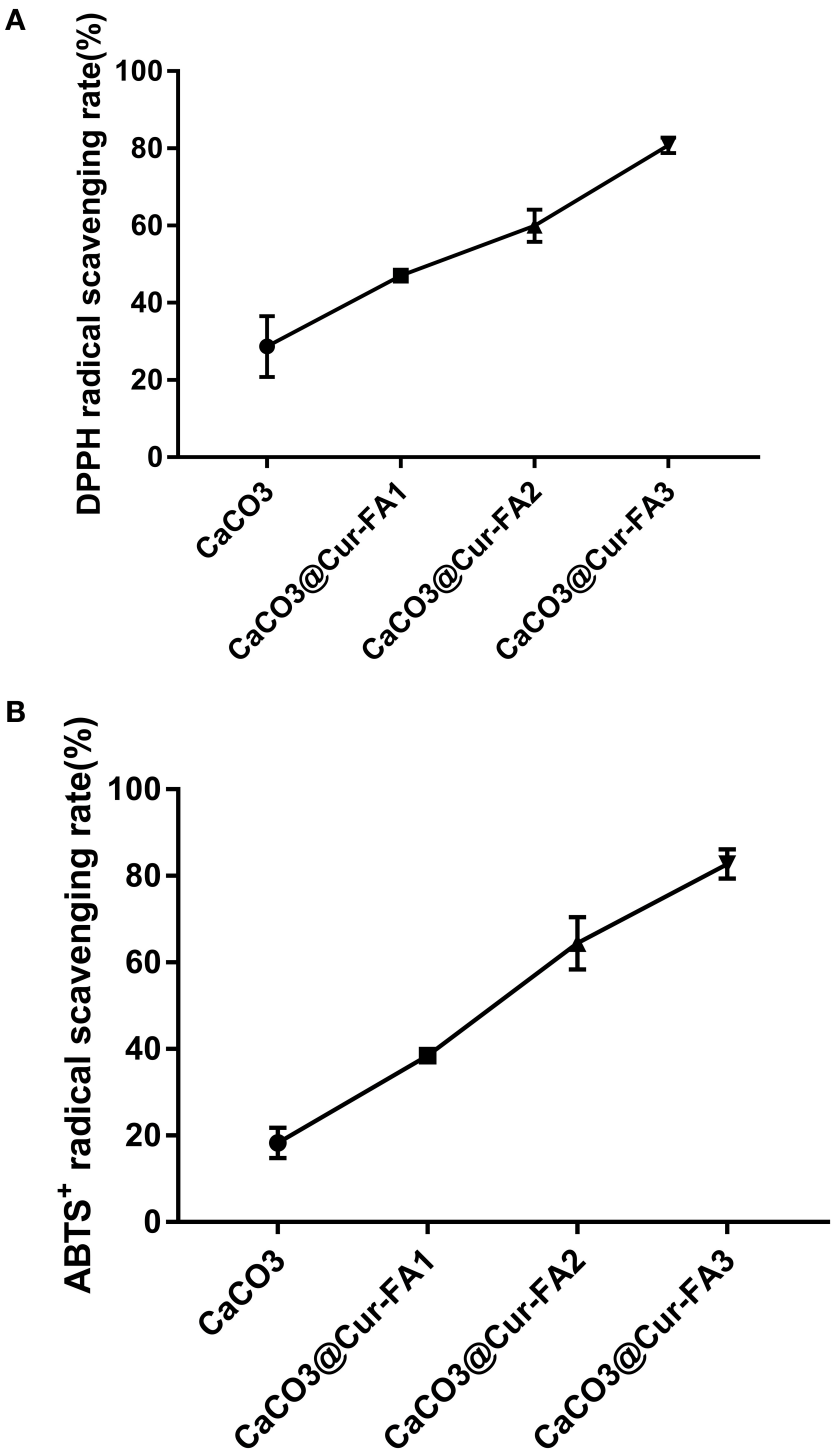
Abilities of CaCO_3_ and CaCO_3_@Cur-FA to scavenge DPPH **(A)** and ABTS^+^ **(B)** radicals.

Antioxidant activity plays a vital role in the food industry. It is the ability of inhibiting or delaying additional molecular oxidation (14). Previous results have demonstrated that Cur (15) and FA (16) have very high antioxidant activity because of phenolic hydroxyl groups in their molecular structure. In our previous study, the antioxidant activity of Cur ester derivatives was shown to be significantly increased. A higher Cur-FA level resulted in the higher antioxidant activity of the Cur-FA film. The DPPH scavenging activity of CaCO_3_@Cur-FA1 microparticles was increased three times compared with that of CaCO_3_ microparticles (Figure 3A). The ABTS^+^ scavenging ability of CaCO3@Cur-FA was similar to the DPPH scavenging ability (Figure 3B). The free radical scavenging ability of CaCO_3_@Cur-FA microparticles was increased by 40% compared with that of CaCO_3_ microparticles. Thus, CaCO_3_@Cur-FA exhibited good antioxidant ability.

### Antimicrobial Activity

CaCO_3_@Cur-FA exhibited antimicrobial activity, which is significant for its application as a food additive (16). In the present work, we adopted the agar–well diffusion method to detect the ability of CaCO_3_@Cur-FA to inhibit *Rhizopus, A. niger, E. coli, and S. aureus*, and the inhibition zones were found to be 5.6 ± 0.3, 13.6 ± 0.3, 14.6 ± 0.2, and 9.3 ± 0.4 mm, respectively (Table 2). Thus, CaCO_3_@Cur-FA inhibited the growth of these pathogens. CaCO_3_@Cur-FA microparticles demonstrated superior antimicrobial activity than CaCO_3_ microparticles, indicating that adding CaCO_3_ promoted the antimicrobial activity of Cur-FA.

**Table 2:** Bacteriostatic activity of films.

**Items**	***Escherichia coli***	***Escherichia coli***	***Aspergillus niger***	***Rhizopus***
CaCO_3_	8.2 ± 0.2^d^	7.1 ± 0.3^c^	10.5 ± 0.4^d^	4.2 ± 0.1^d^
CaCO_3_@Cur-FA1	13.2 ± 0.3^a^	8.3 ± 0.2^b^	12.5 ± 0.6^cd^	4.3 ± 0.5^b^
CaCO_3_@Cur-FA2	14.3 ± 0.4^a^	9.1 ± 0.3^b^	13.1 ± 0.4^b^	5.1 ± 0.4^bc^
CaCO_3_@Cur-FA3	14.6 ± 0.2^a^	9.3 ± 0.4^a^	13.6 ± 0.3^a^	5.6 ± 0.3^a^

Comparison of differences between groups (a, b, c, and d).

## Conclusion

A novel food additive comprising Cur-FA was designed and synthesized in this study. Herein, we successfully engineered a simple, efficient, well-characterized, and stable CaCO_3_@Cur-FA nano-microemulsion as a food additive. The results demonstrated that CaCO_3_@Cur-FA is a natural antimicrobial food additive with excellent antioxidant effects. The antimicrobial activity of CaCO_3_@Cur-FA was irreversibly changed with the change in the environmental pH, and our results demonstrated that CaCO3@Cur-FA could be used as a food additive with a visible pH sensitivity, therefore, it could be used as an antibacterial preservative, or as a protective agent for some drugs that are easy to be damaged by gastric acid. Additionally, CaCO_3_ enhanced the antimicrobial and antioxidant activities of Cur-FA. In conclusion, CaCO_3_@Cur-FA is a natural material with high safety and degradability and may be extensively used as a food additive.

## Data Availability Statement

The original contributions presented in the study are included in the article/supplementary material, further inquiries can be directed to the corresponding authors.

## Author Contributions

LW and ZZ: research concept, methodology, data extraction, analysis, and draft writing. YY: resource searching, verification, formal analysis, supervision, and manuscript reviewing and editing. XW and DL: resources, methodology, project administration, supervision, and manuscript reviewing and editing. ZG: resource searching and manuscript reviewing and editing. Mengying Xia: methodology and manuscript reviewing and editing. All authors reviewed and approved the final manuscript.

## Funding

The present work was funded by the National Natural Science Foundation of China (Grant Nos. 31602098 and 32072906), the Scientific and Technological Project of Henan Province (Grant Nos. 192102110188 and 192102110184), the Postgraduate Research and Practice Innovation Program of Jiangsu Province (Grant No. KYCX20-1494), the Veterinary Drugs Science Subject in Henan University of Animal Husbandry and Economy (Grant No. 41000003), the Scientific Research and Innovation Team in Henan University of Animal Husbandary and Economy (Grant No. 2018KYTD18), and the Science and Technology Major project of Prevention and Treatment of Major Infectious Diseases such as AIDS and Viral Hepatitis: Research on new technology of integrated field rapid detection of important viruses (Grant No. ZX10711001-003-003).

## Conflict of Interest

The authors declare that the research was conducted in the absence of any commercial or financial relationships that could be construed as a potential conflict of interest.

## Publisher's Note

All claims expressed in this article are solely those of the authors and do not necessarily represent those of their affiliated organizations, or those of the publisher, the editors and the reviewers. Any product that may be evaluated in this article, or claim that may be made by its manufacturer, is not guaranteed or endorsed by the publisher.
